# Antioxidant Nobiletin Enhances Oocyte Maturation and Subsequent Embryo Development and Quality

**DOI:** 10.3390/ijms21155340

**Published:** 2020-07-27

**Authors:** Yulia N. Cajas, Karina Cañón-Beltrán, Magdalena Ladrón de Guevara, María G. Millán de la Blanca, Priscila Ramos-Ibeas, Alfonso Gutiérrez-Adán, Dimitrios Rizos, Encina M. González

**Affiliations:** 1 Department of Animal Reproduction, National Institute for Agriculture and Food Research and Technology (INIA), Avda. Puerta de Hierro, 28040 Madrid, Spain; yncajas@gmail.com (Y.N.C.); kecanon@gmail.com (K.C.-B.); magdalenaladron@gmail.com (M.L.d.G.); mgmillanb@gmail.com (M.G.M.d.l.B.); priscilaramosibeas@gmail.com (P.R.-I.); agutierr@inia.es (A.G.-A.); drizos@inia.es (D.R.); 2 Department of Anatomy and Embryology, Veterinary Faculty, Complutense University of Madrid (UCM), 28040 Madrid, Spain

**Keywords:** nobiletin, oocyte quality, antioxidant, ROS, cattle, in vitro

## Abstract

Nobiletin is a polymethoxylated flavonoid isolated from citrus fruits with wide biological effects, including inhibition of reactive oxygen species (ROS) production and cell cycle regulation, important factors for oocyte in vitro maturation (IVM). Therefore, the objective of the present study was to evaluate the antioxidant activity of nobiletin during IVM on matured bovine oocyte quality (nuclear and cytoplasmic maturation; oocyte mitochondrial activity; intracellular ROS and glutathione (GSH) levels) and their developmental competence, steroidogenesis of granulosa cells after maturation, as well as quantitative changes of gene expression in matured oocytes, their cumulus cells, and resulting blastocysts. Bovine cumulus-oocyte complexes were in vitro matured in TCM-199 +10% fetal calf serum (FCS) and 10 ng/mL epidermal growth factor (EGF) (Control) supplemented with 10, 25, 50, or 100 μM of nobiletin (Nob10, Nob25, Nob50, and Nob100, respectively) or 0.1% dimethyl sulfoxide (CDMSO: vehicle for nobiletin dilution). A significantly higher percentage of matured oocytes in metaphase II was observed in Nob25 and Nob50 compared to other groups. Similarly, cleavage rate and cumulative blastocyst yield on Days 7 and 8 were significantly higher for Nob25 and Nob50 groups. Oocytes matured with 25 and 50 μM nobiletin showed a higher rate of migration of cortical granules and mitochondrial activity and a reduction in the ROS and GSH content in comparison with all other groups. This was linked to a modulation in the expression of genes related to metabolism (*CYP51A1*), communication (*GJA1*), apoptosis (*BCL2*), maturation (*BMP15* and *MAPK1*), and oxidative stress (*SOD2* and *CLIC1*). In conclusion, nobiletin offers a novel alternative for counteracting the effects of the increase in the production of ROS during IVM, improves oocyte nuclear and cytoplasmic maturation, and subsequent embryo development and quality in cattle.

## 1. Introduction

In vitro maturation (IVM) of bovine oocytes is one of the most important processes for the development of other assisted reproductive techniques, such as in vitro production of embryos (IVP). The accomplishment of this technique requires successful IVM that involves nuclear, cytoplasmic, and molecular maturation, necessary for subsequent embryonic development [1]. Nevertheless, IVP of cattle embryos still has limitations, considering that not all the oocytes have the ability to develop into a viable embryo after IVM [2], as the culture systems decrease the quality of these gametes [3]. One of the causes is the increase in the production of reactive oxygen species (ROS) caused by the oxygen tension at which IVM is performed [4]. Under normal conditions, the cell produces a ROS level that acts beneficially for tissue regeneration, intracellular redox regulation, and embryogenesis, but an excess of ROS can oxidize cellular molecules, such as lipids, carbohydrates, amino acids, and nucleic acids, modifying their functions and compromising cellular viability by producing lipid peroxidation, mitochondrial damage, and apoptosis [4]. The strategy to avoid the harmful effects caused by an excess of ROS is the use of a wide variety of antioxidants during IVM, such as Vitamin C that protects cells against ROS and acts as an inhibitor of lipid peroxidation [5], cysteamine, which increases intracellular glutathione (GSH) content that protects cells from the deleterious effects of oxidative stress [6] and catalase, which reduces the intracellular concentrations of ROS during IVM and the percentage of apoptotic cells [3]. Moreover, exogenous antioxidants can also act as signaling molecules in steroidogenesis and intracellular redox regulation during IVM [6,7,8]. In recent years, there have been promising results with compounds of natural origin, such as resveratrol [8] or quercetin [6]. However, it is not yet clear which antioxidant is the most efficient to support the development, production, and quality of bovine embryos.

Nobiletin, a class of polymethoxylated flavone identified from the citrus peel (chemically known as 5,6,7,8,3′,4′ hexamethoxyflavone), has drawn increasing attention since it is easily absorbed across the cytoplasmic membranes due to its structure and lipophilic nature [9,10]. Nobiletin interacts with several signaling pathways (ERK, PI3K/AKT, CREB) to promote survival in various cell lines [10,11]. Moreover, nobiletin has a broad range of biological effects, including cell cycle regulation [10], reduction of apoptosis [11,12] and antioxidation [13], important also for the success of oocyte IVM.

Thus, in this study, we aimed to evaluate the antioxidant activity of nobiletin during IVM on matured bovine oocyte quality and their developmental competence. The parameters evaluated were, (i) nuclear (meiotic progression to metaphase II (M-II)) and cytoplasmic maturation (cortical granules (CG) and mitochondrial distribution pattern), (ii) oocyte mitochondrial activity, and intracellular ROS and GSH levels (iii), steroidogenesis of granulosa cells (iv), oocyte developmental competence to blastocyst stage, and (v) quantitative changes of gene expression in matured oocytes, their cumulus cells (CCs) and produced blastocysts.

## 2. Results

### 2.1. Nobiletin Enhances Oocyte In Vitro Maturation and Reduces Oxidative Stress

When evaluating the effect of nobiletin on nuclear maturation, we observed that a concentration of 25 (87.0 ± 0.6%) and 50 µM (89.3 ± 0.4%) increased (*p* < 0.05) the percentage of oocytes reaching M-II compared to all other groups (Nob10: 72.9 ± 0.4%; Nob100: 71.5 ± 0.8%; Control: 71.7 ± 0.8%; and CDMSO: 70.5 ± 0.5%) (Table 1).

**Figure 1:**
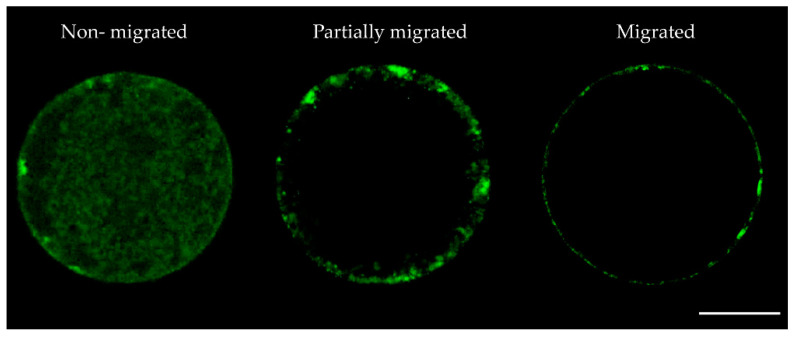
Representative fluorescent images of cortical granules (CG) distribution patterns in bovine oocytes after in vitro maturation in the presence of nobiletin. Scale bar 50 µm.

The migration of CG to the cortical region of the oocyte, as well as mitochondrial distribution and their activity, were used as indicators to analyze cytoplasmic maturation. In the assessment of the cortical granule distribution patterns oocytes matured in the presence of Nob25 (85.7 ± 0.3%) and Nob50 (89.9 ± 2.2%) displayed a higher incidence of migrated CG than oocytes in the Control (69.1 ± 1.1%), CDMSO (69.6 ± 0.9%), Nob10 (72.1 ± 1.0%) and Nob100 (71.2 ± 0.7%) groups (*p* < 0.05). The presence of oocytes with a partially migrated pattern was lower (*p* < 0.05) in Nob25 and Nob50 than all other groups. Similarly, the non-migrated pattern distribution of CG was lower (*p* < 0.05) for nobiletin groups compared to the Control group, while for CDMSO, Nob10 and Nob100 no differences were observed (Table 1). Representative images of CG distribution in matured oocytes are presented in Figure 1.

**Figure 2:**
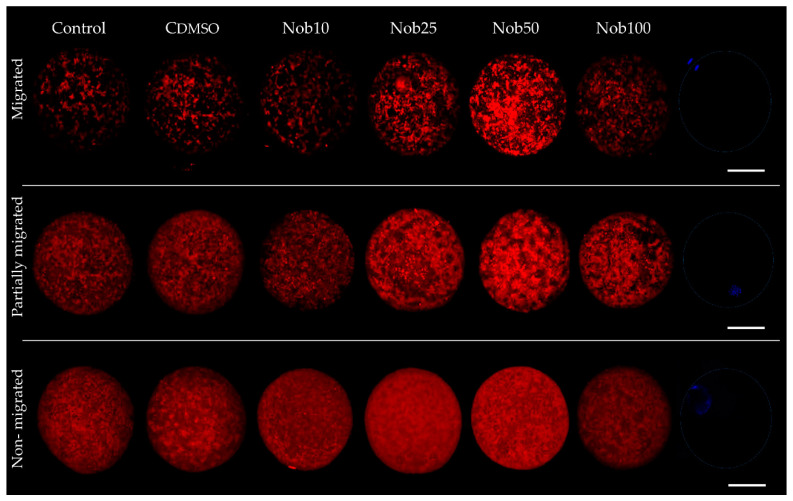
Representative fluorescent images of mitochondria migration pattern in bovine oocytes after in vitro maturation in the presence of nobiletin. Control: oocytes cultured in the presence of synthetic oviductal fluid (SOF) and 5% fetal calf serum (FCS); CDMSO: oocytes cultured in the presence of SOF + 5% FCS supplemented with 0.01% DMSO; Nob10, Nob25, Nob50, Nob100 oocytes cultured in presence of SOF + 5% FCS supplemented with 10, 25, 50 and 100 µM nobiletin, respectively. Scale bar 50 µm.

Regarding the mitochondrial distribution patterns, we found higher migration (*p* < 0.05) in oocytes matured with Nob25 (86.7 ± 0.6%) and Nob50 (88.9 ± 1.2%) compared to Control (71.3 ± 1.5%), CDMSO (69.7 ± 1.0%); Nob10 (73.7 ± 1.0%) and Nob100 (71.6 ± 0.5%) groups. The partially migrated mitochondrial pattern was lower (*p* < 0.05) in the oocytes matured with Nob25 and Nob50 compared to all other groups, while the incidence of non-migrated mitochondria pattern was lower (*p* < 0.05) only for Nob50 group (Table 1). Representative images of mitochondrial distribution in matured oocytes are presented in Figure 2. Quantification of mitochondrial activity in oocytes was measured by fluorescence intensity and a significant increase in intensity was observed in oocytes maturated with Nob25 and Nob50 compared to all other groups (*p* < 0.05; Supplementary Figure S1).

**Figure 3:**
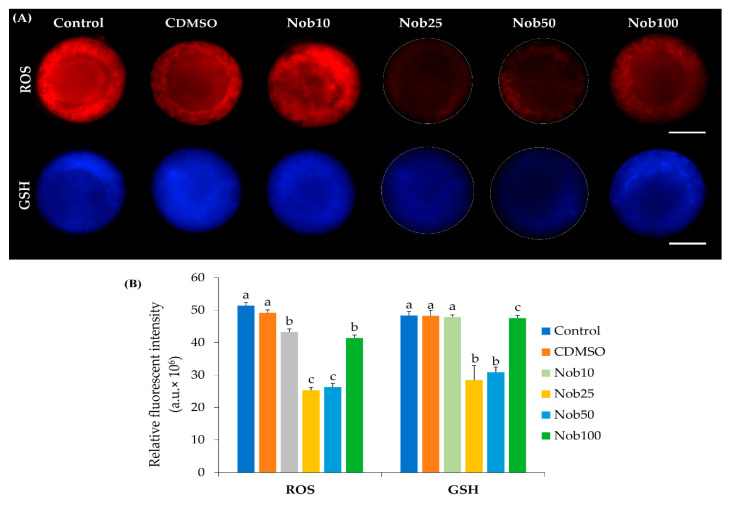
Reactive oxygen species (ROS) and glutathione (GSH) fluorescence intensity in bovine oocytes after in vitro maturation in the presence of nobiletin (**A**) Representative fluorescent images of ROS and GSH fluorescence intensity in bovine oocytes after in vitro maturation in the presence of nobiletin. Control (*n* = 54); CDMSO (*n* = 48); Nob10 (*n* = 50); Nob25 (*n* = 47); Nob50 (*n* = 53); Nob100 (*n* = 49). (**B**) Quantification of relative fluorescent intensity of ROS and GSH in bovine oocytes after in vitro maturation in the presence of nobiletin. Data are the mean ± SEM. Values with different superscript letters differ significantly (*p* < 0.05). Scale bar 50 μm.

When evaluating the effect of nobiletin on oxidative stress, through a relative of ROS and GSH fluorescence intensity in maturated oocytes, we observed that the intensity in both parameters was lower (*p* < 0.05) in Nob25 and Nob50 groups compared with oocytes matured with Nob10 and Nob100 and control groups (Figure 3).

**Figure 4:**
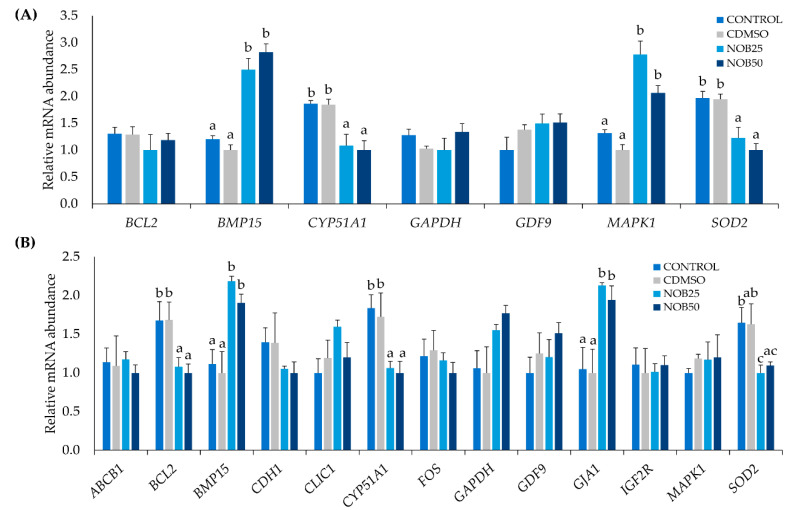
Relative mRNA transcript abundance (normalized against that of the endogenous control H2A histone family member Z (*H2AFZ*) gene and actin beta (*ACTB*)). (**A**) Bovine oocytes after in vitro maturation in the presence of nobiletin. (**B**) Bovine cumulus cells (CCs) after in vitro maturation in the presence of nobiletin. ATP-binding cassette subfamily B member 1 (*ABCB1*), BCL2- apoptosis regulator (*BCL2*), Bone morphogenetic protein 15 (*BMP15*), Cadherin 1 (*CDH1*), Chloride intracellular channel 1 (*CLIC1*), Cytochrome P450, family 51, subfamily A, polypeptide 1 (*CYP51A1*), Fos Proto-oncogene, AP-1 transcription factor subunit (*FOS*), Glyceraldehyde-3-phosphate dehydrogenase (*GAPDH*), Growth differentiation factor 9 (*GDF9*), Gap junction protein alpha 1 (*GJA1*), Insulin like growth factor 2 receptor (*IGF2R*), Mitogen-activated protein kinase 1 (*MAPK*), Superoxide Dismutase 2, Mitochondrial (former MnSOD) (*SOD2*). Data are the mean ± SEM. Different letters above columns indicate significant differences in gene expression among the experimental groups (*p* < 0.05).

Based on these results and to verify the effects of nobiletin on in vitro maturation and oxidative stress we analyzed gene expression in oocytes and their CCs. Only the experimental groups that showed better qualitative parameters in the previous experiments (Nob25and Nob50) were used in comparison with both control groups (Control and CDMSO). Supplementation of IVM medium with nobiletin, irrespective of the concentration, induced the upregulation of *MAPK1* and *BMP15* (developmental-related transcripts) and downregulation of *SOD2* and *CYP51A1* (oxidative stress transcripts) in oocytes after IVM when compared with control groups (*p* < 0.05). No significant differences were observed for the remaining transcripts studied (*BCL2*, *GAPDH*, *GDF9*) (Figure 4A). In CCs, nobiletin produced changes in the expression levels of genes related to quality and development (Figure 4B). *BMP15* (development) and *GJA1* (cell junctions) transcripts were upregulated (*p* < 0.05), while the expression of the oxidative stress (*SOD2, CYP51A1*) and apoptosis (*BCL2*) genes were downregulated in nobiletin groups compared to controls (*p* < 0.05). No significant differences were observed for the remaining transcripts studied (*ABCB1, CDH1, CLIC1, FOS, GAPDH, GDF9, IGF2R,* and *MAPK1*).

### 2.2. Nobiletin Increases Estradiol (E_2_) and Progesterone (P_4_) Production by Cumulus Cells

**Figure 5:**
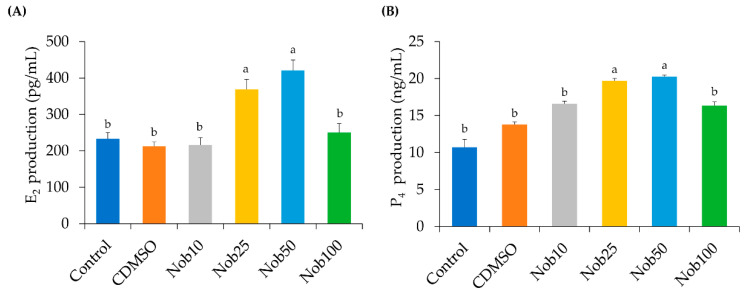
Steroidogenic production of cumulus cells (CCs) after in vitro maturation using different concentrations of nobiletin. (**A**) Steroidogenic production of Estradiol (E_2_). (**B**) Steroidogenic production of Progesterone (P_4_). Bars represent mean concentrations produced by CCs under each different experimental condition. Data are the mean ± SEM. Values with different superscript letters differ significantly (*p* < 0.05).

After IVM, a significant increase in E_2_ production by CCs was found in maturation medium supplemented with Nob25 (368.6 ± 27.3 pg/mL) and Nob50 (421.0 ± 28.2 pg/mL) compared with the rest of the groups (Control: 233.2 ± 16.9 pg/mL; CDMSO: 212.4 ± 11.8 pg/mL; Nob10: 216.2 ± 20.0 pg/mL; and Nob100: 250.2 ± 24.4 pg/mL (*p* < 0.05; Figure 5A). Likewise, a significant increase in P_4_ production by CCs in media after maturation was detected within Nob25 (19.7 ± 0.3 ng/mL) and Nob50 (20.2 ± 0.2 ng/mL) groups compared with the remaining groups (*p* < 0.05; Figure 5B).

### 2.3. Nobiletin Increases Embryo Development and Quality

Embryonic development was assessed after IVM in the presence of nobiletin (Table 2). Cleavage rate and cumulative blastocyst yield at Day 7 and 8 were higher (*p* < 0.05) for Nob25 and Nob50 compared to all other groups. Based on these results, and for blastocysts quality evaluation only the Nob25 and Nob50 groups with both control groups (Control and CDMSO) were used for gene expression analysis.

**Figure 6:**
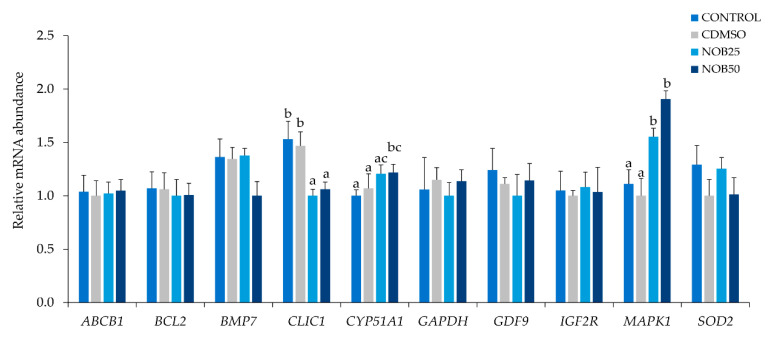
Relative mRNA transcript abundance (normalized against that of the endogenous control H2A histone family member Z (*H2AFZ*) gene and actin beta (*ACTB*)) of blastocysts D7 developed from oocytes matured in the presence of nobiletin. Data are the mean ± SEM. Different letters above columns indicate significant differences in gene expression among the experimental groups (*p* < 0.05).

The expression of *MAPK1* was upregulated, while *CLIC1* was downregulated in blastocysts produced after oocyte maturation with nobiletin supplementation, irrespective of the concentration, compared with blastocysts from control groups (*p* < 0.05). The expression of *CYP51A1* was upregulated in blastocysts from the Nob50 group compared to blastocysts from control groups (*p* < 0.05). No significant differences were observed for the remaining transcripts studied (*ABCB1*, *BCL2*, *BMP7*, *GAPDH*, *GDF9*, *IGF2R*, and *SOD2*) (Figure 6).

## 3. Discussion

Nobiletin, a class of polymethoxylated flavone, has a broad range of biological effects including cell cycle regulation, reduction of apoptosis and antioxidation. To our knowledge, the present study is the first to investigate the effects of nobiletin supplementation in IVM on bovine oocyte quality and their developmental competence. We found that nobiletin while increases steroidogenesis of CCs, it also improves oocyte nuclear and cytoplasmic maturation (mitochondrial activity and CG migration) and decreases oocyte intracellular ROS and GSH levels, reflected to differentially expressed genes related to maturation, metabolism, cell communication, apoptosis and oxidative stress. Furthermore, nobiletin in IVM improves oocyte developmental competence and the quality of produced blastocyst in terms of the expression of genes linked to metabolism, development and oxidative stress.

Cumulus cells play an important role during oocyte growth and maturation, among them supply nutrients [14] and to mediate the effects of hormones during oocyte maturation [15]. Mingoti et al. [16] demonstrated that CCs of bovine COCs can secrete E_2_ and P_4_ in maturation media, and Endo et al. [17] and Sakaguchi et al. [18], demonstrated that exogenous and endogenous E_2_ by granulosa cells directly supports the in vitro development of bovine COCs. In the present study, supplementation with 25 and 50 μM nobiletin in maturation medium increase in E_2_ and P_4_ production by CCs. This is in line with a study by Horigame et al. [19] that demonstrated that nobiletin enhanced testosterone production in cultures of Leydig cells via cAMP/CREB signaling. Therefore, our results indicated that nobiletin might act directly or synergistically with other hormones during oocyte maturation to alter the CCs steroidogenesis in vitro and that the increase of P_4_ and E_2_ production, without any steroid hormone supplementation, plays a positive role in oocyte nuclear and cytoplasmic maturation.

Nuclear maturation was improved by nobiletin supplementation to the IVM medium. This is in line with other studies using different antioxidants, such as resveratrol, astaxanthin or melatonin supplementation in bovine oocyte maturation in vitro [8,20]. However other studies in farm animals using a broad spectrum of antioxidants did not show an effect on the nuclear maturation rate, such as in pigs [5] and cattle [6]. These results suggest that different effects of antioxidants on nuclear maturation could be related to their capacity to activate the mitogen-activated protein kinase 1 (MAPK) pathway since in mammals, MAPK is responsible for meiotic progression [21], and in bovine oocytes, the two main isoforms (ERK1/2) of MAPK are activated near the time of germinal vesicle breakdown (GVBD) [22]. One of the biological effects of nobiletin is the activation of MAPK activity shown in different cell types [10]. Hence, a more plausible explanation for an increase in M-II following nobiletin supplementation could be through P_4_ and stimulation of MAPK1/ERK2, which plays a fundamental role in the regulation of microtubule organization, spindle assembly, chromosome distribution and meiosis resumption [23]. A similar function was demonstrated for resveratrol with improved meiosis resumption by enhancing the expression of Mos/MEK1/p42 MAPK cascade genes [8]. Based on the above, it could be hypothesized that nobiletin regulates the secretion of androgens in the CCs, and improved meiosis resumption by activation of MAPK; however, more experiments are necessary to corroborate this hypothesis.

Next, we observed that supplementation of nobiletin during in vitro maturation improved also cytoplasmic maturation. The migration of CG to the cortical region of the oocyte, as well as mitochondrial distribution and their activity, are suitable indicators to analyze cytoplasmic maturation [24,25]. Hosoe and Shioya [26] and Hoodbhoy et al. [27] demonstrated that proteins released by the CG are also necessary for preimplantation embryo development. We demonstrated that the addition of 25 or 50 µM nobiletin to IVM medium significantly increased peripheral distribution of CG, suggesting that nobiletin at these concentrations could act promoting a better organization of microfilaments and therefore, improving CG migration. A similar effect of CG migration was described with sodium nitroprusside for bovine oocytes in vitro maturation [28].

Mitochondria play an important role since they are a key component of the metabolic machinery responsible for the supply of energy that is consumed during the maturation process [29] and are also the main generator of free radicals in mammals [30]. The movement of mitochondria to areas of high energy consumption is crucial for the oocyte and the embryo during critical periods of the cell cycle. For this reason, the mitochondrial cytoplasmic distribution pattern has been associated with the quality and developmental capacity of mammalian oocytes and embryos [31,32]. We demonstrated that the addition of 25 or 50 µM nobiletin to IVM medium significantly increased mitochondrial migration, giving rise to granular aggregations throughout the cytoplasm in the oocyte after IVM. This pattern of distribution is similar to that described for bovine oocytes in other studies, which demonstrated that mitochondrial reorganization is necessary for cytoplasmic maturation, rearrangement of the cytoskeleton and developmental capacity after IVF [31,33,34]. Another important function of mitochondria is to synthesize adenosine triphosphate (ATP) through β-oxidation and this process involves the electron transport chain [4]. However, electrons may be lost during this process and could be bond to O_2_, resulting in the production of ROS that decreases the developmental competence of the oocyte [35]. Thus, our results of increased oocyte mitochondrial activity could be related to the cytoprotective effects of nobiletin and its intrinsic ROS-scavenging property.

Under normal conditions, cells maintain their ROS levels in equilibrium [6], while during IVM, the cells may suffer disturbances in redox equilibrium having deleterious effects on development [3,36]. However, studies demonstrated that the addition of antioxidants into the culture medium reduces the harmful effects of ROS during IVM and offers a way of protecting the oocyte and subsequent embryo [5,6]. In the oocyte, the main ROS scavenger system is GSH that uses a reducing power provided by oxidative metabolism [3]. Our results show that 25 or 50 μM nobiletin supplementation in IVM medium reduced the intracellular ROS levels, which is in agreement with the use of other flavonoids such as resveratrol [8], quercetin and taxifolin [37] and other class of antioxidants like vitamin C [6]. Regarding nobiletin, studies in cell cultures demonstrated its ability to significantly decrease ROS generation [13] but to date, there are no studies available on its effects in oocytes and embryos. Nobiletin has a beneficial effect on cell protection [10], and like other antioxidants, this effect could be produced due to its hydrophobic nature, which allows it to incorporate into the membrane [37], inhibiting ROS attack and decreasing lipid peroxidation. Therefore, the positive effect observed in the present study could be attributed to this property; nonetheless, further studies are necessary to understand the mechanism of its antioxidant effects in oocytes.

In cytoplasmic maturation, GSH is considered a biochemical marker for oocyte quality, and plays an important role in maintaining redox homeostasis, hence protecting the embryo from oxidative damage before genomic activation [7]. Our results showed decreased levels of GSH in 25 or 50 μM nobiletin supplemented groups, opposed to other studies reporting either a reduction in ROS levels associated with an increase in GSH levels [6,8] or no increase in GSH levels in bovine oocytes [3]. A reasonable explanation for this could be due to the highest mitochondrial activity found in the oocytes from Nob25 and Nob50 groups. It is widely known that increased mitochondrial activity leads to an increase in the exchange of electrons in the inner mitochondrial membrane, which is considered one of the main sources of ROS production [38]. Despite a high mitochondrial activity, the intracellular ROS levels in the mature oocytes from the Nob25 and Nob50 groups were lower than those observed in the oocytes from the control groups, suggesting that GSH was consumed to avoid the harmful effects of the high levels of ROS. This explanation has been proposed before by Rocha-Frigoni et al. [3] for cysteine and cysteamine antioxidant activity during bovine oocyte IVM and by Qu et al. [39] for nobiletin reduction of ROS levels in response to cadmium-induced neuronal injury in rats.

Improvements in oocyte quality by 25 and 50 µM nobiletin supplementation during in vitro maturation were reflected by increased blastocyst development rates on Day 7 and 8. These results are in line with other studies which evaluated other flavonoids like resveratrol [8], or antioxidants such as cysteamine [6], vitamin C [40], lycopene [41], and carnitine [42] in the IVM medium. Furthermore, flavonoids or antioxidants in the IVM showed an interaction with the expression of certain qualitatively related genes to the development of mature oocytes and/or the production of blastocysts.

To test if the effects of nobiletin during IVM were related to gene expression changes, we analyzed the expression of candidate genes for oxidative stress, embryo development, and quality. Superoxide dismutase 2 (*SOD2*), an indicator of oxidative stress [42] was downregulated in oocytes and CCs obtained from Nob25 and Nob50 groups compared with controls, whereas in blastocysts it was not altered. This is in accordance with the findings of Gülcin [43], who showed that superoxide plays an important role in the neutralization of ROS, so a reduction in ROS formation requires less *SOD2* to neutralize free radicals. On the other hand, Chloride intracellular channel 1 (*CLIC1*) is considered as a sensor of cell oxidation [44,45] and is involved in ROS production [45]. Our results showed that *CLIC1* was downregulated in blastocysts obtained from Nob25 and Nob50 groups compared with the controls, both also with increased embryo yield, which agree with earlier studies showing that *CLIC1* expression accompanied by low accumulation of ROS improves embryo development [46]. These findings together with the low intracellular ROS and GSH levels in the oocytes matured with nobiletin supplementation indicate an improvement of their antioxidant activity and consequently an enhanced quality of the produced blastocysts.

Cytochrome P450 family 51 subfamily A polypeptide 1 (*CYP51A1*), Bone morphogenic protein 15 (*BMP15*), Mitogen-activated protein kinase 1 (*MAPK1*), Gap junction alpha-1 protein (*GJA1*) and *BCL2*- apoptosis regulator (*BCL2*), are genes considered quality biomarkers of in vitro matured oocytes [47,48]. *CYP51A1* participates in the regulation of cholesterol biosynthesis [49] and it has been demonstrated that biosynthesis of cholesterol is one example of metabolic cooperation between granulosa cells and oocytes [50]. Furthermore, the upregulation of the enzyme coded by *CYP51A1* is a result of negative feedback reflecting lowered cholesterol availability, which is implicated in the lower quality of oocytes [49]. Therefore, downregulation of *CYP51A1* mRNA expression observed in oocytes and their CCs matured with nobiletin supplementation could be an indicator of good quality. In contrast, 50 μM nobiletin supplementation in IVM upregulated the expression of *CYP51A1* in blastocysts. This is in line with the results of nobiletin supplementation in liver cell culture (HepG2), showing upregulation of CYP1 (Cytochrome P450s family) and improved cholesterol synthesis due to full methoxylation in the A-ring of nobiletin chemical structure [10,51]. Hence, nobiletin could act differently depending on the cell type, probably due to the bioactivity or its chemical structure, which causes that *CYP51A1* might be down or upregulated to control cholesterol availability, however, more in deep studies are necessary to corroborate this information.

In mammals, *BMP15* is known to be involved in oocyte maturation and cholesterol biosynthesis, being specifically expressed in oocytes and acting on CCs, improving oocyte competence, and early embryo development in cattle [52,53]. Several studies reported an increase in *BMP15* transcript during maturation in buffalo [54] and dog [55] oocytes, which are consistent with our findings of an increase in *BMP15* expression in oocytes and their CCs matured with nobiletin supplementation in IVM, related with their improved developmental competence.

The MAPK family plays an important role in bovine oocyte maturation by inducing GVBD [56]. Likewise, *MAPK1* mRNA plays a key role in oocyte maturation by acting on granulosa and CCs in various species including cattle [22] and dogs [55]. Our results demonstrated that *MAPK1* mRNA expression in oocytes and embryos was upregulated, suggesting that nobiletin could act on cell cycle regulation as reported by Yoshimizu et al. [57] and Morley et al. [58] in other types of cells. On the other hand, *GJA1,* also known as connexin 43 (Cx43), is a component of gap junctions expressed in CCs and a major mediator of cell-to-cell communication via gap junctions, and a proliferation regulator [59]. Recently, it was shown that CCs of bovine oocytes with higher developmental competence express higher *GJA1* [60]. These findings are in agreement with our results demonstrating higher *GJA1* expression in the CCs from oocytes matured with nobiletin. Taken together, these results suggest that nobiletin modifies the expression of key genes for oocyte cytoplasmic development and maturation, improving their developmental competence and increasing embryo yield.

Moreover, we observed that during IVM, nobiletin decreased the expression of *BCL2* in CCs. The downregulation of *BCL2* expression is associated with a protective effect and has been reported to have a critical role in CCs by acting as a regulator of apoptosis [61]. Studies in cattle showed that lycopene (antioxidant) supplementation during in vitro maturation, increases expression of *BCL2* exerting a pro-apoptotic effect [41]. Studies that used nobiletin on human cancer cell lines (gastric, hepatic, and breast) shown that nobiletin induced apoptotic cell death by reducing the expression of *BCL2* [10,12,58]. However, the molecular mechanisms whereby nobiletin induces apoptosis among different carcinogenic cells remain poorly understood. Therefore, it is to be assumed that nobiletin has different actions for healthy and unhealthy cells.

In conclusion, a concentration 25 or 50 μM nobiletin offers a novel alternative for counteracting the effects of the increase in the production of ROS during IVM and subsequent embryo development in cattle. In matured oocytes and their cumulus cells, nobiletin modifies the expression of genes involved in maturation (*BMP15* and *MAPK1*), metabolism (*CYP51A1*), communication (*GJA1*), apoptosis (*BCL2*) and oxidative stress (*SOD2* and *CLIC1*), which was reflected in the increased nuclear and cytoplasmic maturation (mitochondrial activity and CG migration) and CCs steroidogenesis, decreased intracellular ROS and GSH levels, as well as enhanced embryo development and quality. These benefits of nobiletin can be attributed to its bioactivity, chemical structure, and antioxidant properties, and might be a tool to overcome ROS disorders in bovine IVP embryos and to improve ART in mammals.

## 4. Materials and Methods

Unless stated otherwise, all chemicals were purchased from Sigma-Aldrich Corporation (St Louis, MO, USA).

### 4.1. Oocyte Collection and In Vitro Maturation

Immature cumulus-oocyte complexes (COCs) were obtained by aspirating follicles (2–8 mm diameter) from the ovaries of mature heifers (i.e., at least one corpus luteum or remained scars from previous ovulations in one or both ovaries) collected at local slaughterhouses. A total of 3758 class 1 and 2 COCs (homogeneous cytoplasm and intact CCs) were matured in groups of 50 COCs per well for 24 h, at 38.5 °C under an atmosphere of 5% CO_2_ in air, with maximum humidity [2] in 500 µL of maturation medium, TCM-199 with 10% (*v/v*) fetal calf serum (FCS) and 10 ng/mL epidermal growth factor (Control, *n* = 595); supplemented either with 10, 25, 50, and 100 µM nobiletin (MedChemExpress, MCE, Sollentuna, Sweden); (Nob10, *n* = 645; Nob25, *n* = 630; Nob50, *n* = 603; and Nob100, *n* = 672, respectively) or dimethyl sulfoxide (DMSO control (CDMSO), 0.01% DMSO vehicle for nobiletin dilution, *n* = 613). The concentration of nobiletin was based on the findings of other studies in which this polymethoxylated flavonoid was used in vivo in zebrafish and chick embryos and in vitro in human umbilical vein endothelial cells, showing an anti-angiogenic activity at concentrations between 30 and 100 µM [10,39,62].

After 24 h of IVM, a representative number of matured COCs under different conditions were employed to evaluate: nuclear maturation, cortical granules migration (CG), mitochondria (Mt) distribution patterns and mitochondrial activity, levels of ROS and GSH and mRNA abundance of selected genes (oocytes and their CCs). The remaining oocytes were processed for in vitro fertilization and culture to assess their developmental competence. To analyze the mRNA abundance of selected genes, four pools of 10 matured COCs were collected from each treatment, and CCs were physically separated from oocytes by gentle pipetting in phosphate-buffered saline (PBS). Oocytes, in pools of 10 per treatment group, were washed in PBS, snap-frozen in liquid N_2_ (LN_2_), and stored at −80 °C until mRNA extraction. Their corresponding CCs were also washed in PBS, centrifuged at 10,000 g, and then snap-frozen in LN_2_ and stored at −80 °C until mRNA extraction.

To measure the steroidogenic production of COCs after IVM, media from all groups were collected and stored at −20 °C until analysis.

### 4.2. Cortical Granules (CG) Distribution Patterns

Visualization of CG distribution was performed according to Arias-Álvarez et al. [63], with minor modifications. Briefly, in vitro matured COCs from each treatment were first suspended in 100 µL of PBS without calcium or magnesium supplemented with 0.1% polyvinylpyrrolidone (PVP) and their CCs were removed by gentle pipetting. Next, oocytes were treated with 0.5% (*w/v*) pronase to digest the zona pellucida. Zona-free oocytes were washed in PBS + 0.1% PVP three times and fixed in 4% (*w/v*) buffered neutral paraformaldehyde (PF) solution (pH 7.2–7.4) for 30 min at room temperature and then treated with permeabilization solution (0.02% *v/v* Triton X-100 in PBS + 1% Bovine Serum Albumin (BSA) for 10 min). The oocytes were then treated for 30 min with blocking solution (7.5% *w/v* BSA in PBS) and incubated in 100 µg/mL FITC-labeled Lens culinaris (LCA-FITC, Vector Laboratories, Burlingame, CA, USA) for 30 min at room temperature in a dark chamber. Following, oocytes were treated for 30 min with Hoechst 33342 (10 μg/mL) to evaluate nuclear maturation. After staining, oocytes were washed in PBS + 0.1% PVP, mounted in 3.8 μL of mounting medium (50% *v/v* PBS, 50% *v/v* glycerol, 0.5 µg/mL Hoechst) between a coverslip and a glass slide and sealed with nail polish. Slides were examined using a laser-scanning confocal microscope (Leica TCS SP2; Leica Microsystems GmbH, Wetzlar, Germany) equipped with an argon laser excited at 488 nm and whose detection spectrum is 515 nm.

As a measure of cytoplasmic maturation, CG distribution was analyzed (Control: *n* = 58; CDMSO: *n* = 66; Nob10: *n* = 72; Nob25: *n* = 70; Nob50: *n* = 78; Nob100: *n* = 70) and classified as: non-migrated (CGs distributed throughout the cytoplasm); partially migrated (CGs dispersed and partly clustered throughout the cortical area); and migrated (small CG arranged at the periphery or adjacent to the plasma membrane) [26,29]. Simultaneously, oocytes were evaluated for nuclear maturation.

### 4.3. Mitochondrial Distribution Patterns and Quantification of Mitochondrial Activity

Briefly, in vitro matured COCs from each treatment were first suspended in 100 µL PBS + 0.1% PVP and their CCs were removed by gentle pipetting. Next, oocytes were equilibrated for 15 min in maturation medium and then placed in four-well culture plates containing 500 μL of 400 nM MitoTracker DeepRed (Molecular Probes Inc., Eugene, OR, USA) per well. The plates were incubated at 38.5 °C, 5% CO_2_ in the dark, and humidified atmosphere for 30 min. The stained oocytes were washed twice in PBS + 0.1% PVP and fixed in 4% PF for 30 min at room temperature. Following, oocytes were treated for 30 min with Hoechst 33342 (10 μg/mL) for evaluating nuclear maturation. After that, oocytes were washed in PBS + 0.1% PVP, mounted in 3.8 μL of mounting medium between a coverslip and a glass slide and sealed with nail polish. Slides were examined using a laser-scanning confocal microscope (Leica TCS SP2) equipped with an argon laser excited at 644 nm with a detection spectrum of 625–665 nm. The format, laser, gain, and offset were kept constant for every sample. Serial sections of 5 µm were made for each oocyte and a maximum projection was accomplished for each.

Mitochondrial patterns and mitochondrial activity were analyzed in matured oocytes from Control: *n* = 59; CDMSO: *n* = 56; Nob10: *n* = 61; Nob25: *n* = 76; Nob50: *n* = 71; Nob100: *n* = 74. The distribution was classified as: non-migrated (when mitochondria were homogeneously distributed throughout the cytoplasm); partially migrated (mitochondria were heterogeneously distributed throughout the cytoplasm) and migrated (mitochondria were distributed with granular aggregations) [1,29,33,38]. For the assessment of mitochondrial activity, the fluorescence signal intensity (pixels) was quantified. Images obtained were evaluated using the ImageJ program (NIH, ImageJ version 1.52k software (http://rsbweb.nih.gov/ij/), using the freehand selection tool. Fluorescence intensity in each oocyte was determined using the following formula: Relative fluorescence = integrated density (IntDen) − (area of selected oocyte x mean fluorescence of background readings). Fluorescence intensities are expressed in arbitrary units (a.u.) [3,46]. Simultaneously, these oocytes were evaluated for nuclear maturation.

### 4.4. Assessment of Oocyte Nuclear Maturation

Matured oocytes from all treatments stained for CG distribution and mitochondrial distribution and activity were also stained with Hoechst 33342 solution (10 μg/mL of PBS) for nuclear chromosomal and polar body evaluation (Control: *n* = 117; CDMSO: *n* = 122; Nob10: *n* = 133; Nob25: *n* = 146; Nob50: *n* = 149; Nob100: *n* = 144). Oocytes were classified as follows: immature oocytes comprising the stages of germinal vesicle (GV, nucleus well defined), germinal vesicle breakdown (GVBD, chromosome condensation), metaphase I (MI, first metaphasic plate visible); and matured oocytes comprising the stage of metaphase-II (M-II, represented by the presence of the first polar body and/or the second metaphasic plate). Nuclear maturation was assessed under an epifluorescence microscope (Nikon 141731, Tokyo, Japan) equipped with a fluorescent lamp (Nikon HB-10104AF) and UV-1 filter. Oocytes in M-II were considered as matured.

### 4.5. Levels of Reactive Oxygen Species (ROS) and Glutathione (GSH)

For evaluation of ROS and GSH, in vitro matured COCs from each treatment (Control: *n* = 54; CDMSO: *n* = 48; Nob10: *n* = 50; Nob25: *n* = 47; Nob50: *n* = 53; Nob100: *n* = 49), were first suspended in 100 µL PBS + 0.1% PVP and their CCs were removed by gentle pipetting, then were incubated in four-well plates containing 500 μL of 10 µM of CellROX Deep Red Reagent (Invitrogen, Eugene, OR, USA) for ROS and 20 µM of CellTracker Fluorescent (Molecular Probes, Eugene, OR, USA) for GSH per well, at 38.5 °C, 5% CO_2_ in a dark and humidified atmosphere for 30 min. After staining, oocytes were washed twice with PBS+ 0.1% PVP, mounted in 3.8 μL of mounting medium between a coverslip and a glass slide, sealed with nail polish, and were imaged immediately using an epifluorescence microscope (Nikon 141731). Fluorescence emitted from the oocytes was captured using B-2E/C (ROS) and UV-2A (GSH) filters for ten seconds after exposure to UV light. The digital images were processed and analyzed using ImageJ. The relative ROS and GSH fluorescence intensity in each oocyte were assessed as described for the mitochondrial activity (Section 4.3.).

### 4.6. Steroidogenic Production of Estradiol and Progesterone by CCs

Progesterone (P_4_) concentration was measured in spent maturation media by solid-phase radioimmunoassay method (RIA) using the methods as described by Santiago-Moreno et al. [64]. Aliquots of 100 µl were used in duplicate, then each of the samples was measured in the liquid Scintillation Counter (Tri-Carb® 2100TR) including the measurement of the standard curve. The intra-assay coefficient of variation was 11% and assay sensitivity was 0.4 ng/mL. Estradiol (E_2_) concentrations in spent maturation media were measured by a solid phase enzyme-linked immunosorbent assay (ELISA), based on the principle of competitive binding specific kit (DEH3355 DEMEDITEC Diagnostics GmbH, Kiel, Germany) according to the manufacturer´s instructions. Intra-assay coefficients of variation were 6%. Results are expressed as average E_2_ (pg/mL) and P_4_ (ng/mL) concentrations produced by 50 COCs after the IVM period using 3 replicates.

### 4.7. Sperm Preparation and In Vitro Fertilization (IVF)

IVF was performed as described previously [65]. Briefly frozen semen straws (0.25 mL) from an Asturian Valley bull previously tested for IVF were thawed at 37 °C in a water bath for 1 min and centrifuged for 10 min at 280 g through a gradient of 1 mL of 40% and 1 mL of 80% Bovipure (Nidacon Laboratories AB, Göthenborg, Sweden), according to the manufacturer´s instructions. The sperm pellet was isolated and washed in 3 mL of Boviwash (Nidacon Laboratories AB, Göthenborg, Sweden) by centrifugation at 280 *g* for 5 min. The pellet was re-suspended in the remaining 300 µL of Boviwash. The final concentration of spermatozoa was adjusted to 1 × 10^6^ spermatozoa/mL. Gametes were co-incubated for 18–22 h in 500 µL fertilization media (Tyrode’s medium) with 25 mM bicarbonate, 22 mM sodium lactate, 1 mM sodium pyruvate, and 6 mg/mL fatty acid-free bovine serum albumin (BSA) supplemented with 10 mg/mL heparin sodium salt (Calbiochem) in four-well cell culture plates in groups of 50 COCs per well under an atmosphere of 5% CO_2_ in the air, with maximum humidity at 38.5 °C.

### 4.8. In Vitro Culture of Presumptive Zygotes

At 18–22 h post-insemination (hpi), presumptive zygotes from each experimental group (Control: *n* = 359; CDMSO: *n* = 378; Nob10: *n* = 397; Nob25: *n* = 372; Nob50: *n* = 336; Nob100: *n* = 414) were denuded of CCs by vortexing for 3 min and then cultured in groups of 25 in 25 µL droplets of culture medium (synthetic oviductal fluid (SOF) [66]); with 4.2 mM sodium lactate, 0.73 mM sodium pyruvate, 30 µL/mL BME amino acids, 10 µL/mL minimum essential medium (MEM) amino acids and 1 µg/mL phenol red supplemented with 5% FCS under mineral oil at 38.5 °C under an atmosphere of 5% CO_2_, 5% O_2_ and 90% N_2_ with maximum humidity. Cleavage rate was recorded at day 2 (48 hpi) and cumulative blastocyst yield was determined on Days 7 and 8 pi. Pools of ten Day 7 expanding blastocysts from each treatment group were washed in PBS, snap-frozen in LN_2_, and stored at −80 °C until mRNA extraction.

### 4.9. Gene Expression Analysis

Gene expression analysis was performed using four pools of 10 oocytes, and their corresponding CCs and four pools of 10 Day 7 expanded blastocysts per treatment group. All samples were washed in PBS, snap-frozen in LN_2_, and stored at −80 °C until mRNA extraction analyses.

Poly(A) RNA was extracted using the Dynabeads mRNA Direct Extraction Kit (Ambion; Thermo Fisher Scientific Inc., Oslo, Norway) with minor modifications [67]. Immediately after poly(A) RNA extraction, reverse transcription (RT) was performed using an Moloney murine leukemia virus (MMLV) Reverse Transcriptase 1st-Strand cDNA Synthesis Kit according to the manufacturer’s instructions (Epicentre Technologies Corp., Madison, WI, USA). Tubes were heated to 70 °C for 5 min to denature the secondary RNA structure, allowing Poly(T) random primers and Oligo dT annealing, and the RT mix was then completed by adding 0.375 mM dNTPs (Biotools, Madrid, Spain), 6.25 U RNAsin RNAse inhibitor (Promega, Madison, WI, USA), MMLV HP RT 10x reaction buffer, 5 mM DTT and 5 U MMLV high-performance reverse transcriptase. Samples were incubated at 25 °C for 10 min, and then at 37 °C for 60 min, to allow the RT of RNA, and finally at 85 °C for 5 min to denature the enzyme. All mRNA transcripts were quantified in duplicate using a Rotorgene 6000 Real-Time Cycler (Corbett Research, Sydney, Australia). RT–quantitative polymerase chain reaction (qPCR) was performed by adding a 2 µL aliquot of each cDNA sample (~60 ng µL^−1^) to the PCR mix (GoTaq qPCR Master Mix, Promega, Madrid, Spain) containing specific primers to amplify the genes of interest. Primer sequences are provided in Supplementary Table S1. The selection of genes to be evaluated in oocytes, CCs and blastocysts was carried out considering that they are representative of key processes, i.e., communication (*GJA1*), oxidative stress (*SOD2, GAPDH*), metabolism (*CYP51A1),* quality (*BCL2, GDF9, IGF2R*) and development (*BMP15, CLIC1, ABCB1, BMP7, MAPK1, CDH1*) as previously described by [40,46]. All primers were designed using Primer-BLAST software (http://www.ncbi.nlm.nih.gov/tools/primer-blast/) to span exon-exon boundaries when possible. For quantification, RT-qPCR was performed as described previously [68]. The PCR conditions were tested to achieve efficiencies close to 1. Relative expression levels were quantified by the comparative cycle threshold (CT) method [69]. Values were normalized using two housekeeping (HK) genes: *H2AFZ* and *ACTB.* Fluorescence was acquired in each cycle to determine the threshold cycle or the cycle during the log-linear phase of the reaction at which fluorescence increased above background for each sample. Within this region of the amplification curve, a difference of one cycle is equivalent to a doubling of the amplified PCR product. According to the comparative CT method, the ΔCT value was determined by subtracting the mean CT value of the two housekeeping genes from the CT value of the gene of interest in the same sample. The calculation of ΔΔCT involved using the highest treatment ΔCT value (i.e., the treatment with the lowest target expression) as an arbitrary constant to subtract from all other ΔCT sample values. Fold-changes in the relative gene expression of the target were determined using the formula 2^−ΔΔCT^.

### 4.10. Statistical Analysis

All statistical tests were performed using the software package SigmaStat (Systat Software Inc., San Jose, CA, USA). Nuclear maturation, CG and mitochondrial distribution patterns, mitochondrial activity, ROS, and GSH measurements, steroidogenic production of estradiol and progesterone, cleavage and blastocysts rates and relative mRNA abundance were normally distributed with homogeneous variance, so one-way analysis of variance (ANOVA), followed by Tukey´s test, was performed to evaluate the significance of differences between groups. Values were considered significantly different at *p* < 0.05. Unless otherwise indicated, data are presented as the mean ± SEM.

## Figures and Tables

**Table 1:** In vitro maturation of bovine oocytes in the presence of the nobiletin.

Parameters Evaluated	Control	CDMSO	Nob10	Nob25	Nob50	Nob100
Nuclear maturation *n*	117	122	133	146	149	144
Matured (M-II) *n* (%)	84 (71.7 ± 0.8)^b^	86 (70.5 ± 0.5)^b^	97 (72.9 ± 0.4)^b^	127 (87.0 ± 0.6)^a^	133 (89.3 ± 0.4)^a^	103 (71.5 ± 0.8)^b^
Immature *n* (%)	33 (28.2 ± 0.7)^a^	36 (29.5 ± 0.5)^a^	36 (27.1 ± 0.4)^a^	19 (12.9 ± 0.6)^b^	16 (10.7 ± 0.4)^b^	41 (28.4 ± 0.8)^a^
Cytoplasmic Maturation						
Cortical Granules Distribution						
*n*	58	66	72	70	78	70
Migrated *n* (%)	40 (69.1 ± 1.1)^b^	46 (69.6 ± 0.9)^b^	52 (72.1 ± 1.0)^b^	60 (85.7 ± 0.3)^a^	70 (89.9 ± 2.2)^a^	50 (71.2 ± 0.7)^b^
Partially migrated *n* (%)	10 (17.2 ± 2.6)^a^	12 (18.2 ± 1.7)^a^	15 (20.9 ± 0.7)^a^	7 (9.9 ± 1.6)^b^	7 (8.8 ± 1.3)^b^	15 (21.5 ± 0.6)^a^
Non-migrated *n* (%)	8 (13.7 ± 1.9)^a^	8 (12.2 ± 2.0)^ac^	5 (6.9 ± 0.2)^bc^	3 (4.4 ± 1.8)^b^	1 (1.2 ± 1.2)^b^	5 (7.3 ± 0.2)^bc^
Mitochondrial Distribution						
*n*	59	56	61	76	71	74
Migrated *n* (%)	42 (71.3 ± 1.5)^b^	39 (69.7 ± 1.0)^b^	45 (73.7 ± 1.0)^b^	66 (86.7 ± 0.6)^a^	63 (88.9 ± 1.2)^a^	53 (71.6 ± 0.5)^b^
Partially migrated *n* (%)	10 (17.0 ± 0.5)^a^	11 (19.6 ± 1.1)^a^	11 (17.9 ± 1.0)^a^	5 (6.7 ± 0.3)^b^	7 (9.8 ± 1.5) ^b^	13 (17.5 ± 1.5)^a^
Non-migrated *n* (%)	7 (11.7 ± 1.8)^a^	6 (10.8 ± 1.5)^a^	5 (8.3 ± 0.4)^a^	5 (6.6 ± 0.3)^ab^	1 (1.3 ± 1.3)^b^	8 (10.8 ± 1.7)^a^

*n*: number of oocytes assigned per group. Control: oocytes cultured in the presence of synthetic oviductal fluid (SOF) and 5% fetal calf serum (FCS); CDMSO: oocytes cultured in the presence of SOF + 5% FCS supplemented with 0.01% DMSO; Nob10, Nob25, Nob50, Nob100 oocytes cultured in presence of SOF + 5% FCS supplemented with 10, 25, 50, and 100 µM nobiletin, respectively. Data are the mean ± SEM. Within lanes, values with different superscript letters differ significantly (*p* < 0.05).

**Table 2:** Effect of nobiletin on in vitro maturation of bovine oocytes and subsequent embryonic development.

Groups	Total No. Presumptive Zygotes in Culture	Cleavage Rate	Blastocyst Yield	
			Day 7	Day 8
		*n* (%)	*n* (%)	*n* (%)
Control	359	267 (74.2 ± 0.4)^b^	76 (21.1 ± 0.4)^b^	92 (25.8 ± 0.5)^b^
CDMSO	378	278 (73.6 ± 0.5)^b^	78 (20.9 ± 0.4)^b^	98 (26.1 ± 0.7)^b^
Nob10	397	300 (75.6 ±0.3)^b^	75 (18.9 ± 0.4)^b^	90 (23.1 ± 0.7)^b^
Nob25	372	335 (89.9 ± 0.4)^a^	90 (24.4 ± 0.5)^a^	119 (32.2 ± 0.8)^a^
Nob50	336	307 (91.3 ± 0.3)^a^	86 (25.7 ± 0.6)^a^	117 (35.3 ± 0.8)^a^
Nob100	414	306 (74.0 ± 0.6)^b^	76 (18.9 ± 0.9)^b^	100 (24.5 ± 1.0)^b^

*n*: number of oocytes assigned per group. Control: blastocysts cultured in the presence of SOF and 5% FCS; CDMSO: blastocysts cultured in the presence of SOF + 5% FCS supplemented with 0.01% DMSO; Nob10, Nob25, Nob50, Nob100 oocytes cultured in presence of SOF + 5% FCS supplemented with 10, 25, 50 and 100 µM nobiletin, respectively. Data are the mean ± SEM. Within columns, values with different superscript letters differ significantly (*p* < 0.05).

## Supplementary Materials

Supplementary materials can be found at https://www.mdpi.com/1422-0067/21/15/5340/s1. Figure S1: Quantification of mitochondria activity in bovine oocytes after in vitro maturation in the presence of nobiletin. Control (*n* = 59): oocytes cultured in the presence of SOF and 5% FCS; CDMSO (*n* = 56): oocytes cultured in the presence of SOF + 5% FCS supplemented with 0.01% DMSO; Nob10 (*n* = 61), Nob25 (*n* = 76), Nob50 (*n* = 71), Nob100 (*n* = 74) oocytes cultured in presence of SOF + 5% FCS supplemented with 10, 25, 50 and 100 µM nobiletin, respectively, Table S1: Summary of primer sequences used for RT-qPCR in oocytes, CCs and blastocysts after in vitro maturation in the presence of nobiletin.
