# Supplementary material for: Antioxidant Nobiletin Enhances Oocyte Maturation and Subsequent Embryo Development and Quality
Source: Int J Mol Sci. 2020 Jul 27;21(15):5340. doi: 10.3390/ijms21155340 (PMC7432792; doi:10.3390/ijms21155340)
Supplement: Supplementary file 1 [file ijms-21-05340-s001.zip › Supplementary Files_Cajas et al/Table S1_Cajas et al..docx]

Table S1. Primers used for RT-qPCR.

|  | **Gene name** | **Primer sequence (5’- 3’)** | **Fragment size (bp)** | **GenBank accession number** |
| --- | --- | --- | --- | --- |
| *ABCB1* | ATP-binding cassette subfamily B member 1 | F: GAAACGAAGTTAAGATCTTGAAGGGCC  R: CCGTCGATACTGACCATGCCC | 153 | XM_024991021 |
| *ACTB* | Actin, beta | F: GAGAAGCTCTGCTACGTCG  R: CCAGACAGCACCGTGTTGG | 264 | AF191490.1 |
| *BCL2* | BCL2- apoptosis regulator | F: TGGAGCAGGTGCCTCAGGA  R: ATCTCGAAGGAAGTCCAGCGTC | 300 | NM_001166486.1 |
| *BMP7* | Bone morphogenetic protein 7 | F: AACCATGCCATCGTGCAGACGC  R: AAGCCCGGACAACCATGTTTGC | 250 | NM_001206015.1 |
| *BMP15* | Bone morphogenetic protein 15 | F: ATCATGCCATCATCCAGAACC  R: TAAGGGACACAGGAAGGCTGA | 72 | NM_001031752.1 |
| *CDH1* | Cadherin 1 | F: GATTGCAAGTTCCCGCCATC  R: ACATTGTCCCGGGTGTCATC | 144 | NM_001002763 |
| *CLIC1* | Chloride intracellular channel 1 | F: CCATTCCGGATGTGTTTCGTGG  R: GAAACCACCCAGGGCCTTTGTG | 196 | NM_001015608.1 |
| *CYP51A1* | Cytochrome P450, family 51, subfamily A, polypeptide 1 | F: GGCCCAAGGTGATTTCCATTTC  R: CTCCCAAGAAACCCTGCACTGG | 168 | BC149346.1 |
| *FOS* | Fos Proto-oncogene, AP-1 transcription factor subunit | F: CTCTCCTACTACCACTCACC  R: GTACTAGCCATTGTAGGTCC | 153 | NM_182786.2 |
| *GAPDH* | Glyceraldehyde-3-phosphate dehydrogenase | F: ACCCAGAAGACTGTGGATGG  R: ATGCCTGCTTCACCACCTTC | 247 | BC102589 |
| *GDF9* | Growth differentiation factor 9 | F: AGCGCCCTCACTGCTTCTATAT  R: TTCCTTTTAGGGTGGAGGGAA | 80 | NM_174681.2 |
| *GJA1* | Gap junction protein alpha 1 | F: TGCCTTTCGTTGTAACACTCA  R: AGAACACATGAGCCAGGTACA | 142 | NM_174068.2 |
| *H2AFZ* | H2A.Z variant histone 1 | F: AGGACGACTAGCCATGGACGTGTG  R: CCACCACCAGCAATTGTAGCCTTG | 209 | NM_174809 |
| *IGF2R* | Insulin like growth factor 2 receptor | F: GCTGCGGTGTGCCAAGTGAAAAAG  R: AGCCCCTCTGCCGTTGTTACCT | 201 | NM_174352.2 |
| *MAPK1* | Mitogen-activated protein kinase 1 | F: GTCGCCATCAAGAAAATCAGC  R: GGAAGGTTTGAGGTCACGGT | 308 | NM_175793 |
| *SOD2* | Superoxide Dismutase 2, Mitochondrial (former MnSOD) | F: GCTTACAGATTGCTGCTTGT  R: AAGGTAATAAGCATGCTCCC | 101 | S67818.1 |
