# Supplementary figures and images for: Antioxidant Nobiletin Enhances Oocyte Maturation and Subsequent Embryo Development and Quality
Source: Int J Mol Sci. 2020 Jul 27;21(15):5340. doi: 10.3390/ijms21155340 (PMC7432792; doi:10.3390/ijms21155340)

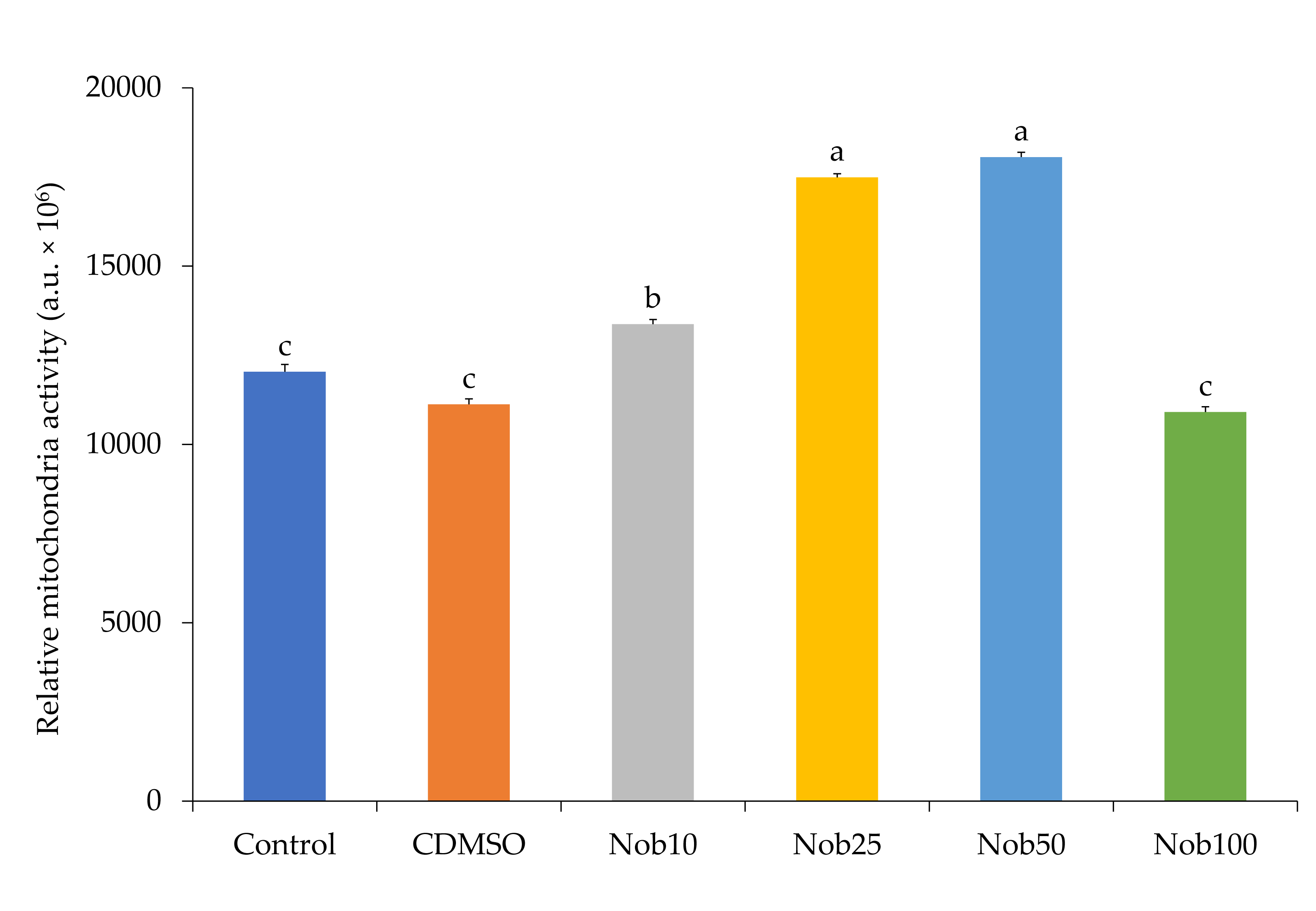

Supplement: Supplementary file 1 [file ijms-21-05340-s001.zip › Supplementary Files_Cajas et al/Revised_Figure S1_Cajas et al.tif]
